# Two Cases of Double Vagina

**Published:** 1847-01

**Authors:** 


*Two Cases of Double Vagina.* By Professor Meigs.—On the — October, 1846, I was called to Mrs. ———, aged 20 years, in labor of her first child. She is a remarkably well formed and comely wowan.

The pains were sharp and frequent, evidently of the kind called dolores præparantes, or grinding pains. After some time, as they had become more violent, I examined the state of the os uteri, which was of the size of a half-dollar, the head of the child presenting, and the ovum unruptured. In the course of an hour more, I examined again, and the os uteri was then nearly dilated. While pressing the palp of my index finger to the left side of the pelvis, it caught in a seeming bridle, which at the instant made me fear the cervix uteri had been broken, so as to detach a semi-circular portion of the os uteri, for the pains had been exceeding sharp, and their returns had been announced by violent cries. It was but a moment that I indulged the idea of a rupture of the cervix, for upon pushing the index farther, and flexing the finger, I found I could draw the point of it outwards, pulling along with it the bridle in question. Still I did not understand the case until, having withdrawn the indicator, I examined with it the structure of the external parts, and then learned that the lady was possessed of a double vagina. Supposing that such a revelation would not be agreeable to her, I kept my own counsel, hoping that the child's head would come down through the right or the left channel without injuring the septum. But after the head escaped from the circle of the os uteri, the bridle or partition would not go definitely to the right or to the left, although I thrust it first one way and then the other. The tie was so strong that the fleshy septum extending from the anterior to the posterior columna of the vagina would not admit of the dilatation of the lower or outer third of the tube. And as the lady was very strong, and had powerful uterine pains, I began to perceive some danger of the vagina being ruptured by the vain efforts for expulsion.

I now explained to the monthly nurse, and to a relative of my patient, the cause of the delay, and the necessity that had arisen. I therefore procured the requisite permission to expose the parts to an inspection. Upon this, the two orifices of the vagina were seen to be exactly alike, and the partition stretched across the head from front to rear of the passage, which by it was wholly prevented from dilating.

I now, with a strong scissors, divided the wall by a single stroke of the instrument, whereupon the child's head advanced, dilated the os magnum, and was speedily delivered with safety to both the mother and her infant. She never complained afterwards relative to the operation, and within a month I met her on foot in the streets.

A week later I was called to a lady in her 30th year, in labor of her first child. Upon examining the state of the os uteri, I found the circle not much bigger than a quarter dollar, with thin margin, and within it the penis of the child; the scrotum being detected within the os uteri after the pain ceased. As it was night, I went to another apartment and slept an hour, when being called, I found the os uteri very much dilated, and a buttock, near which was the right foot, presenting

While inquiring into the state of the cervix, I hooked my finger into a bridle, just as I had done in the case above mentioned, and I confess that the same thought was obvious to me, viz: that she had broken off a half ring of the circle of the os uteri, but I immediately afterwards discovered that I had another case of double vagina under management. In this case the partition was very firm and thick, extending from the os magnum almost up to the os tincæ. I inspected the external structures, and the two vaginas were each perfect and alike, included within labia pudendi common to both.

I was glad to find that only one foot of the child would come down, being fearful that if both should descend, I might not readily prevent one from entering the right and the other the left vagina.

I now disengaged the right foot and brought it down the right channel, the left leg was flexed upon the belly and thorax of the fœtus. With a little assistance the foot was delivered and the buttock of the child coming downwards, thrust the vaginal wall to the left, and so the trunk was delivered. I had great difficulty to extricate the head of the child, which remained long in the vagina; the infant breathing from time to time the air that I admitted through the hollow of my hand and fingers to its mouth and nostrils. The child, a male, was alive and in good health; the mother is quite well recovered.

Some years ago I was called by the late venerable Dr. Ruan to consultation upon a case of double vagina in a primiparous woman. I delivered the child, with the forceps, through the right canal, without difficulty or any injury, and had some five weeks later an inspection of the parts, which, as I remember, were very similar to those described in my second case above.—*Med. Examiner.*

